# HER2-Positive Circulating Tumor Cells in Breast Cancer

**DOI:** 10.1371/journal.pone.0015624

**Published:** 2011-01-10

**Authors:** Michail Ignatiadis, Françoise Rothé, Carole Chaboteaux, Virginie Durbecq, Ghizlane Rouas, Carmen Criscitiello, Jessica Metallo, Naima Kheddoumi, Sandeep K. Singhal, Stefan Michiels, Isabelle Veys, José Rossari, Denis Larsimont, Birgit Carly, Marta Pestrin, Silvia Bessi, Frédéric Buxant, Fabienne Liebens, Martine Piccart, Christos Sotiriou

**Affiliations:** 1 Department of Medical Oncology, Institut Jules Bordet, Brussels, Belgium; 2 Breast Cancer Translational Research Laboratory, Institut Jules Bordet, Brussels, Belgium; 3 Department of Surgical Oncology and Gynecology, Institut Jules Bordet, Brussels, Belgium; 4 Department of Pathology, Institut Jules Bordet, Brussels, Belgium; 5 Department of Gynecology, Hôpital St Pierre, Brussels, Belgium; 6 Translational Research Unit, Hospital of Prato, Prato, Italy; 7 Department of Gynecology, Hôpital Erasme, Brussels, Belgium; Health Canada, Canada

## Abstract

**Purpose:**

Circulating Tumor Cells (CTCs) detection and phenotyping are currently evaluated in Breast Cancer (BC). Tumor cell dissemination has been suggested to occur early in BC progression. To interrogate dissemination in BC, we studied CTCs and HER2 expression on CTCs across the spectrum of BC staging.

**Methods:**

Spiking experiments with 6 BC cell lines were performed and blood samples from healthy women and women with BC were analyzed for HER2-positive CTCs using the CellSearch®.

**Results:**

Based on BC cell lines experiments, HER2-positive CTCs were defined as CTCs with HER2 immunofluoresence intensity that was at least 2.5 times higher than the background. No HER2-positive CTC was detected in 42 women without BC (95% confidence interval (CI) 0–8.4%) whereas 4.1% (95%CI 1.4–11.4%) of 73 patients with ductal/lobular carcinoma in situ (DCIS/LCIS) had 1 HER2-positive CTC/22.5 mL, 7.9%, (95%CI 4.1–14.9%) of 101 women with non metastatic (M0) BC had ≥1 HER2-positive CTC/22.5 mL (median 1 cell, range 1–3 cells) and 35.9% (95%CI 22.7–51.9%) of 39 patients with metastatic BC had ≥1 HER2-positive CTC/7.5 mL (median 1.5 cells, range 1–42 cells). In CTC-positive women with DCIS/LCIS or M0 BC, HER2-positive CTCs were more commonly detected in HER2-positive (5 of 5 women) than HER2-negative BC (5 of 12 women) (p = 0.03).

**Conclusion:**

HER2-positive CTCs were detected in DCIS/LCIS or M0 BC irrespective of the primary tumor HER2 status. Nevertheless, their presence was more common in women with HER2-positive disease. Monitoring of HER2 expression on CTCs might be useful in trials with anti-HER2 therapies.

## Introduction

Minimal residual disease (MRD) after primary surgery is thought to contribute to disease relapse in early breast cancer (BC) [1]; [2]. The presence of peripheral blood Circulating Tumor Cells (CTCs) is considered as a surrogate marker for the evaluation of MRD. CTCs are epithelial tumor cells detected in the peripheral blood of patients with solid tumors using mainly cytometric/antibody-based and molecular approaches [1]; [2]. Currently, CellSearch® is the only technology for CTC detection that has been cleared by the Food and Drug administration (FDA) as an aid in the monitoring of patients with metastatic breast, colorectal and prostate cancers. The detection of ≥5 CTCs/7.5 mL of peripheral blood before starting a new treatment in metastatic BC was associated with worse clinical outcome [3]. Data for CTC detection and phenotyping in early BC by CellSearch® are only now emerging. In the German Success trial, ≥1 CTC/23 mL of peripheral blood were detected in 20% and 21% of approximately 1500 patients with early BC before or after adjuvant chemotherapy, respectively [Rack B, et al. 2010 J Clin Oncol 28:7s; suppl; abstr 1003]. Two multicenter neoadjuvant studies (Remagus II and GeparQuattro) and two single center studies have used CellSearch® for CTC detection in early BC [4]–[7]. In the Remagus II study, detection of ≥1 CTC/7.5 mL before, after neoadjuvant chemotherapy or at both time points was associated with worse distant metastasis free survival (DMFS) and overall survival (OS) at a median follow-up of 36 months [8]. In the GeparQuattro trial, HER2 overexpressing CTCs were observed in 14 of 58 CTC-positive cases (24.1%) including 8 patients with HER2-negative primary tumors and 3 patients after neoadjuvant trastuzumab. Several groups have reported the detection of bone marrow HER2-positive DTCs or peripheral blood HER2-positive CTCs in BC using different detection methods [9]–[15].

In a BALB-NeuT mice model, Husemann et al demonstrated that cytokeratin positive (CK+) cells and HER2 expressing cells (HER2+) became detectable in bone marrow at as early as 4–9 weeks of age when the most detailed analysis of the mammary gland could only detect areas of atypical ductal hyperplasia (ADH) [16]. The same investigators reported the detection of CK+ tumor cells in the bone marrow of 13% of 39 women with DCIS [16]. Based on these findings, Husemann et al suggested that systemic spread is an early step in BC progression [16].

To interrogate tumor cell dissemination during BC progression, we sought to detect CTCs and study the expression of HER2 on CTCs across the spectrum of BC staging: ductal/lobular carcinoma in situ, (DCIS/LCIS), early invasive (M0) and metastatic (M1) BC. To this aim, we used the CellSearch® technology (Veridex, New Jersey, USA), a semi-automated system for the detection and HER2 phenotypic profiling of CTCs. At the same time, we developed a method to quantify HER2 expression on CTCs.

## Methods

### Patients

Women with DCIS/LCIS, M0 and M1 BC treated at the Institut Jules Bordet (IJB), Hôpital Saint Pierre and Hôpital Universitaire Erasme in Belgium between November 2007 and June 2009 were screened. Characteristics of the patients included in this study are presented in Table 1. All patients received locoregional and systemic treatment according to local institutional practice. The blood draw was performed in women with DCIS/LCIS 3 weeks after primary surgery up to 5 years after primary diagnosis, in women with M0 BC either before the initiation of (neo) adjuvant chemotherapy or during the follow-up period up to 5 years from the initial diagnosis and in women with M1 BC either before starting a new treatment for progressive disease or while receiving treatment for stable or responding disease. Women with a second BC were excluded from the analysis unless the two lesions were diagnosed within a year or the two lesions had concordant HER2 status. Every patient and healthy women included in this study signed a written informed consent. This study was approved by the Ethics Committee of the Institut Jules Bordet (reference CE1468), the Ethics Committee of Centre Hospitalier Universitaire Saint Pierre (reference AK/08-04-3575/27) and the Ethics Committee of Université Libre de Bruxelles (ULB)- Hôpital Erasme (reference P2007/303).

**Table 1:** Patient characteristics.

	DCIS/LCIS(N = 73)	M0 Breast Cancer(N = 101)	M1 Breast Cancer(N = 39)
**Age**			
≤50	19	49	13
>50	52	52	26
Unknown	2		
**ER**			
Positive	50	84	34
Negative	15	17	5
Unknown	8		
**PR**			
Positive	46	66	26
Negative	19	35	13
Unknown	8		
**HER**			
Positive	19	19	6
Negative	33	81	33
Unknown	21	1	
**Histology Grade**			
1	9	10	2
2	12	49	20
3	42	36	13
Unknown	10	6	4

M0: Non-metastatic Breast Cancer, M1: Metastatic Breast Cancer, DCIS: Ductal Carcinoma in situ, LCIS: Lobular Carcinoma in situ, ER: Estrogen receptor, PR: Progesterone Receptor, HER2: Erbb2.

For healthy women, women with DCIS/LCIS and M0 BC patients, HER2-positive CTCs were detected using a modified ficoll procedure to reduce the 22.5 mL of peripheral blood draw to 7.5 mL that could then be processed on the CellSearch®. This step was performed in order to increase the sensitivity of the technique for earlier stages of the disease. For patients with M1 disease, 7.5 mL of blood were drawn from each patient to detect HER2-positive CTCs using the CellSearch®. The first 5 mL of blood obtained in this study was always discarded to avoid epithelial cell contamination from the skin.

### HER2-positive CTC detection using the CellSearch®

Peripheral blood samples were enriched for cells expressing the epithelial-cell adhesion molecule (EPCAM) with antibody-coated magnetic beads, and cells were labeled with the fluorescent nucleic acid dye 4,2-diamidino-2-phenylindole dihydrochloride. Fluorescently labeled monoclonal antibodies specific for leukocytes (CD45–allophycocyan) and epithelial cells (cytokeratin 8,18,19–phycoerythrin) were used to distinguish epithelial cells from leukocytes. Moreover, the CellSearch® Tumor Phenotyping Reagent HER2 (a fluorescein conjugated monoclonal antibody) was used in conjunction with the CellSearch® Epithelial Cell Kit to phenotype CTCs for the presence of HER2. The identification and enumeration of CTCs was performed using the CellSearch® Analyzer, a semi-automated fluorescence-based microscopy system that permits computer-generated reconstruction of cellular images [17]; [18]. CTCs were defined as nucleated cells lacking CD45 and expressing cytokeratin [17]; [18]. Quality control was maintained via the CellSearch® CTC Control Kit used to standardize reagents, instruments, and operator technique.

HER2 expression on CTCs using CellSearch® was calibrated through peripheral blood spiking experiments with MCF7, ZR75-1, BT20, MDA MB361, SKBR3 and BT474 cell lines. Briefly, 22.5 mL of peripheral blood from a healthy donor were spiked with approximately 500 cells from these cell lines and then processed by a modified ficoll procedure and the CellSearch® using the same protocol described above for patients with localized disease. The intensity of HER2 staining of CTCs from patients with BC observed on the FITC channel of the CellSearch® system was a continuous variable ranging from absent to very weak, weak, intermediate, bright and very bright staining. Riethdorf et al [5] proposed a model in which HER2 staining of CTC by CellSearch® was classified as 0, 1+, 2+, 3+. We sought to develop a method to quantify HER2 expression on CTCs. To that aim, we assessed HER2 intensity on CTCs using a formula that calculates how many times the HER2 staining intensity of the cell is higher from background:
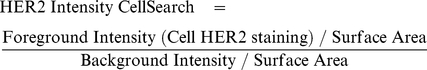



The Foreground Intensity (Cell HER2 staining)/Surface Area and the Background Intensity/Surface Area have been calculated using the “Aida Image Analyzer v3.45” software. The reproducibility of this method to quantify HER2 expression on CTCs was assessed by 3 independent readers that quantified the *HER2 Intensity CellSearch®* of 30 CTCs.

Representative CellSearch® images of 15 cells from each cell line were analyzed and the median HER2 Intensity for each cell line was calculated. In addition, HER2 gene status for the above cell lines was analyzed by fluorescence *in situ* hybridization (FISH), (Pathvysion, Abbott-Vysis®) (60 nuclei were analyzed per cell line). HER2 protein expression was assessed by immunocytochemistry (ICC) on slides spotted with 5000 cells from each cell line. *HER2 Intensity CellSearch®* was correlated with the results from FISH and ICC for HER2.

### Independent Blind Central Image Review by Veridex

For CellSearch®, source of variability between different labs has been considered the inter-reader variability [19]. This becomes more important in early BC where CTCs are present in very low numbers [19]. Therefore, in order to assure the quality of our results, an independent blind review of the entire set of CellSearch® images from 86 cases of healthy women and women with BC was performed by Pharma Testing Services, CellSearch®, Veridex US. Only events that met stringent criteria for CTC definition by CellSearch® were considered as positive. Results of the review were discussed between IJB investigators and Veridex. A review of all remaining cases was performed by IJB investigators using the same stringent criteria for CTC definition by CellSearch®.

### Primary tumor characteristics

The Elston-Ellis modification of the Scarff-Bloom-Richardson (SBR) grading system (Nottingham grading system) was used to define histological grade [20]. Estrogen and progesterone receptor (ER and PR) were scored based on Harvey et al [21] and Leake et al [22]. HER2 status of the primary tumor was determined according to the American Society of Clinical Oncology/College of American Pathologists Guideline Recommendations using immunohistochemistry and FISH [23].

### Statistical analysis

Associations between categorical variables were assessed using the chi-square test. The Mann-Whitney test was used to compare continuous variables between 2 groups. Pearson correlation was used to assess correlation between continuous variables. Analyses were performed using SPSS (SPSS Inc. Chicago, IL) version 15.0. Results are reported according to the REMARK guidelines [24].

## Results

### CTCs in Breast Cancer

As a control group, we studied 36 healthy women and 6 women operated for atypical ductal/lobular hyperplasia (ADH/ALH). The detection rate in the control group was 3/42 or 7.1% (95%CI 2.5–19.0%, median 1 cell, range 1–2 cells). In 73 women with DCIS/LCIS, ≥1 CTC/22.5 mL of blood was observed in 6/73 or 8.2% (95%CI 3.8–16.7%, median 1 cell, range 1–2 cells). In 101 women with M0 BC, we observed ≥1 CTC/22.5 mL in 11.9% (95%CI 5.7–18.3%, median 1 cell, range 1–4 cells). No association was observed between patient characteristics and detection of ≥1 CTC/22.5 mL in M0 breast cancer (Table 2). Finally, among 39 women with metastatic BC, we detected ≥1 CTC/7.5 mL of blood in 23/39 or 59% (95%CI 43.4–72.9%, median 9 cells, range 1–640 cells).

**Table 2:** CTCs detection and patient characteristics in non metastatic invasive (M0) breast cancer.

	All patients	≥1 CTCs/22.5 mL	
	N	N (%)	P-value
**Age**			
≤50	49	6 (12)	0.91
>50	52	6 (11)	
**ER**			
Positive	84	8 (9)	0.10
Negative	17	4 (23)	
**PR**			
Positive	66	7 (11)	0.59
Negative	35	5 (14)	
**HER**			
Positive	19	4 (21)	0.18
Negative	81	8 (10)	
**Histology Grade**			
1	10	2 (20)	0.22
2	49	3 (6)	
3	36	6 (17)	
**(Neo) adjuvant Chemotherapy**			
Blood draw before	65	9 (14)	0.41
Blood draw after	36	3 (8)	

ER: Estrogen receptor, PR: Progesterone Receptor, HER2: Erbb2.

### HER2 staining on CTCs

**Figure 1:**
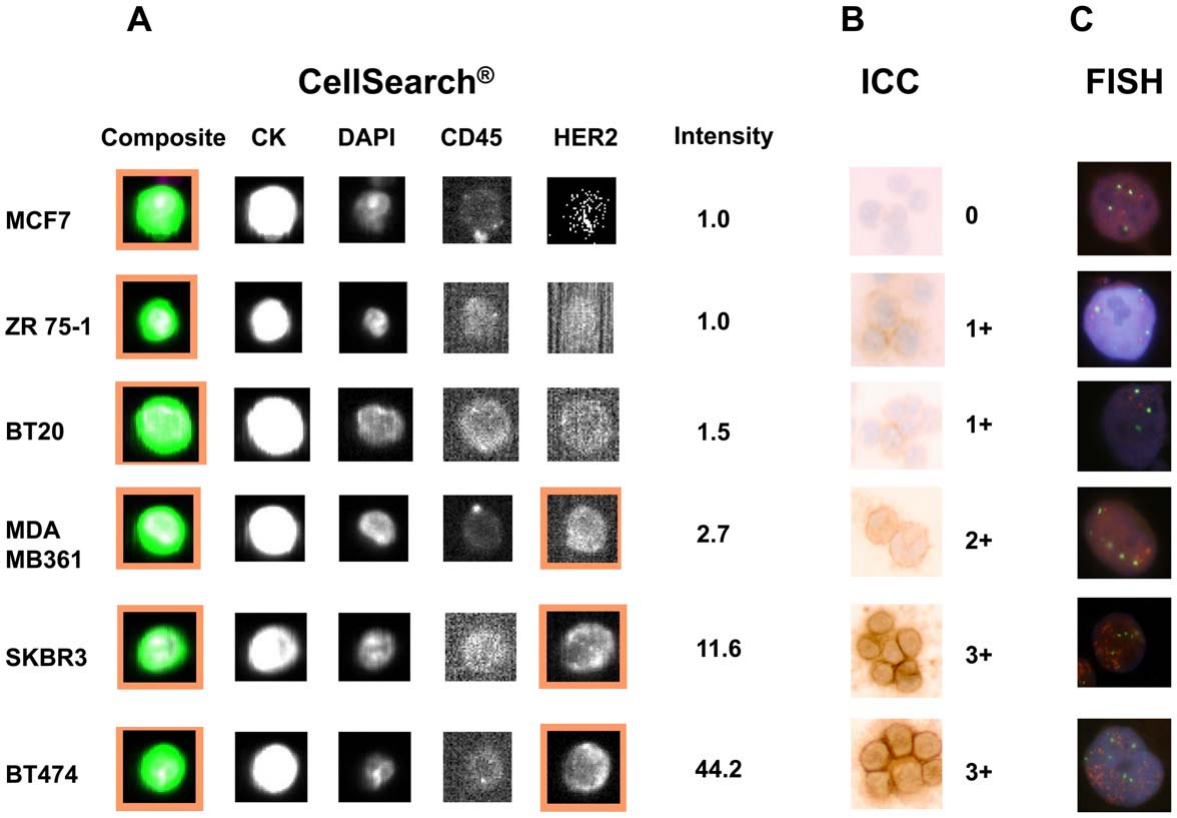
HER2 protein expression and gene amplification in 6 breast cancer cell lines. After spiking experiments with these cell lines, HER2 protein expression was evaluated using the CellSearch® technology (FITC-labeled anti-HER2 antibody) and intensity of HER2 immunofluorecence were evaluated using the *HER2 Intensity CellSearch®* formula as described in the methods. In the same cell lines, HER2 protein expression was assessed by ICC (B) and HER2 gene status by FISH (C).

HER2 expression of 6 BC cell lines was evaluated using CellSearch® and *HER2 Intensity CellSearch®* for each cell was calculated as described in the methods. In the same cell lines, HER2 gene status was assessed by FISH and HER2 protein expression by ICC (Figure 1A, B, C). Median *HER2 Intensity CellSearch®* in these cell lines correlated both with the results of the FISH for HER2 gene (pearson correlation 0.92, p = 0.03) and ICC for HER2 protein (pearson correlation 0.89, p = 0.04) (Table 3).

**Table 3:** *HER2 Intensity CellSearch®* (HER2 protein), Immunocytochemistry, ICC (HER2 protein), fluorescence in situ hybridization, FISH (HER2 gene) in 6 breast cancer cell lines.

Cell Line	*HER2 Intensity CellSearch*®(HER2 protein)		ICC(HER2 protein)	FISH(HER2 gene)
	Median	Range	Scoring	HER2/CEP17 FISH Ratio
**MCF7**	1.0	1.0–2.0	0	1.0
**ZR75-1**	1.0	1.0–1.8	1+	1.2
**BT20**	1.0	1.0–2.2	1+	1.8
**MDA MB361**	3.1	1.0–11.2	2+	2.9
**SKBR3**	11.6	4.7–256.8	3+	4.2
**BT474**	39.3	4.2–175.5	3+	5.9

*HER2 Intensity CellSearch®* can be used as a continuous variable. In order to assess the reproducibility of our method to quantify HER2 expression on CTCs, 3 independent readers quantified the *HER2 Intensity CellSearch®* of 30 CTCs (Table S1). We observed more than 99% correlation in *HER2 Intensity CellSearch®* between the independent readers (pearson correlation reader 1 vs 2: 0.99, p<0.001, reader 1 vs 3: 1.00, p<0.001, reader 2 vs 3: 1.00, p<0.001).

**Figure 2:**
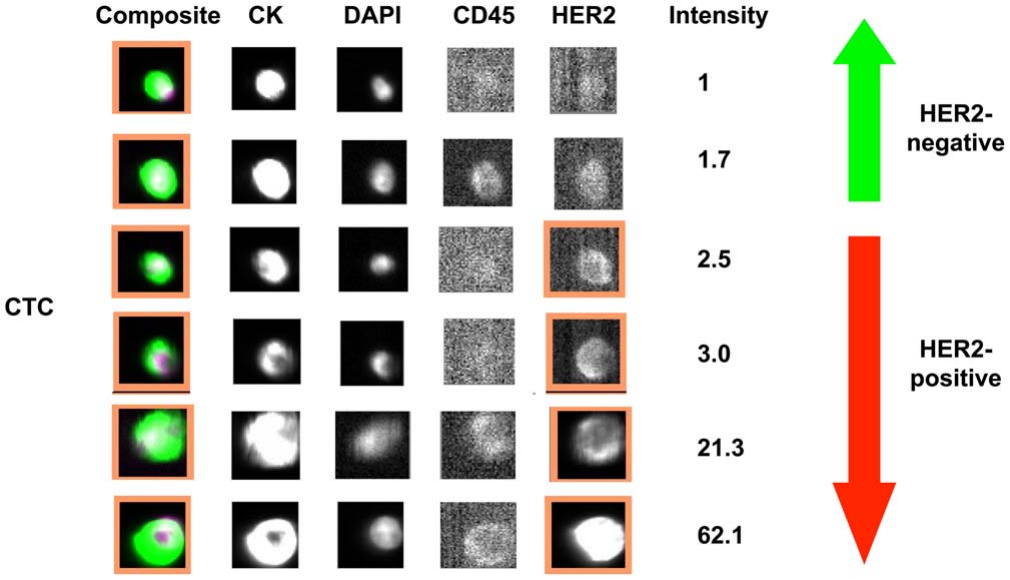
CellSearch® images of CTCs from patients with breast cancer. HER2-positive CTCs were considered as CTCs having *HER2 Intensity CellSearch®* ≥2.5.

Depending on the biological and clinical research question, different cut offs can be also used in order to define HER2-positive CTCs. A higher cut off can define CTCs with HER2 protein over expression/HER2 gene amplification in a more specific but less sensitive way than a lower cut off. We fixed a cut off for *HER2 Intensity CellSearch®* that is at least 2.5 times higher than the background so that all MCF7, ZR75-1 and BT20 cells (cell lines without HER2 amplification) were considered HER2-negative by CellSearch®, whereas two third (67%) of MDA MB361 (cell line with moderate HER2 amplification) and all SKBR3 and BT474 cells (cell lines with strong HER2 amplification) were considered HER2-positive. Therefore, in the present study we considered a CTC as HER2-positive by CellSearch® if there was a HER2 staining in the FITC channel compatible with a cell morphology and a *HER2 Intensity CellSearch®* that was at least 2.5 times higher than the background (Figure 2, Table S2).

### HER2-positive CTCs in Breast Cancer

No HER2-positive CTC was observed in any of the 36 healthy women or the 6 women operated for ADH/ALH (95%CI 0–8.4%). Interestingly, 3 of 73 patients with DCIS/LCIS had 1 HER2-positive CTC/22.5 mL (4.1%, 95%CI 1.4–11.4%) and 8 of 101 patients with M0 BC had ≥1 HER2-positive CTC/22.5 mL (7.9%, 95%CI 4.1–14.9%, median 1 cell, range 1–3 cells). Among women with DCIS/LCIS or M0 BC and detectable CTCs, 5 of 5 women with HER2-positive tumors versus 5 out of 12 women with HER2-negative primary tumors had ≥1 HER2-positive CTC (p = 0.03).

Among 39 women with M1 BC, ≥1 HER2-positive CTC/7.5 mL were detected in 14/39 or 35.9% (95%CI 22.7–51.9%, median 1.5 cells, range 1–42 cells). Among 33 women with M1 BC and HER2-negative primary tumor, ≥1 HER2-positive CTC/7.5 mL were detected in 39.4%, (95%CI 24.7–56.3%, median 1.5 cells, range 1–42 cells).

## Discussion

In the present study ≥1 CTC/22.5 mL of blood were detected in 7.1% of women without BC, 8.2% of women with DCIS/LCIS and 11.9% of women with M0 BC. Pierga et al. detected ≥1 CTC/7.5 mL in 23% of 97 patients before the administration of neoadjuvant chemotherapy and in 17% of 86 patients after the administration of neoadjuvant chemotherapy [4]. Riedthorf et al. detected ≥1 CTC/7.5 mL in 21.6% of 213 patients before neoadjuvant treatment (NT) and in 10.6% of 207 patients after NT [5]. Sandri et al. detected one to three CTCs/30 mL in approximately 30% of 56 patients with localized BC before surgery [7]. Lang et al. detected ≥1 CTC/30 mL of blood in 38% of 92 patients [6]. In the SUCCESS study, ≥1 CTC/23 mL of peripheral blood were detected in 20% and 21% of patients before or after adjuvant chemotherapy, respectively in a cohort of approximately 1500 patients with early BC [Rack B, et al. 2010 J Clin Oncol 28:7s; suppl; abstr 1003]. In the above studies, blood draws were performed before primary surgery with the exception of the SUCCESS study in which blood draws were performed 3 weeks after surgery and at the end of chemotherapy. The lower CTC detection rates in M0 BC in our series could be due: (1) to the fact that blood draw was not performed before primary surgery but instead 3 weeks after surgery up to 5 years from the diagnosis, (2) a step of ficoll enrichment was performed which is known to lead to cell loss [25], (3) only cells meeting the stringent criteria for CTC definition by CellSearch® were considered positive after an independent blind review by Veridex. Although a direct comparison between different studies cannot be performed, the CTC detection rate when analyzing 22.5 mL of blood by CellSearch® after a modified ficoll procedure in our study was not higher than the CTC detection in 7.5 mL of blood by Pierga et al [4] and Riedthorf et al [5]. Our results question the utility of applying a modified ficoll procedure, before CellSearch®, which may additionally increase complexity and raise issues of reproducibility among laboratories.

We have developed the *HER2 Intensity CellSearch®* formula which allows for the quantification of HER2 immunofluoresence intensity by CellSearch®. By performing spiking experiments using 6 BC cell lines, we propose that HER2-positive CTCs can be considered as those having *HER2 Intensity CellSearch®* that is at least 2.5 higher than the background. The clinical utility of defining HER2-positive CTCs based on the above cut off should be prospectively tested. In this study, we have detected no HER2-positive CTC in 42 women without BC, ≥1 HER2-positive CTC/22.5 mL in 4.1% of women with DCIS/LCIS and 7.9% of women with M0 BC. The detection of HER2-positive CTCs in women with DCIS/LCIS is in line with the early dissemination model of BC metastasis suggested by Husemann et al [16]. Further studies are needed to validate these results. We have reported the detection of HER2-positive CTCs in women with HER2-negative early BC as other investigators using CellSearch® or different technologies [5]; [9]–[11]. In our series of non metastatic BC, HER2-positive CTCs were more commonly detected in HER2-positive as opposed to HER2-negative primary tumors but these results should be interpreted with caution due to the low number of cases with HER2-positive CTCs. Furthermore, as reported by other investigators [12]–[15] we have detected HER2-positive CTCs in women with metastatic BC and initially HER2-negative primary tumors.

The role of CTCs or HER2-positive CTCs in metastatic BC is currently evaluated in clinical trials. Example of such a trial is the Southwest Oncology Group (SWOG) trial (ClinicalTrials.gov NCT00382018) which is testing the strategy of changing chemotherapy compared with continuing the same treatment for metastatic BC patients with elevated CTCs levels at the first follow-up assessment. Another example is the study of lapatinib in advanced BC patients with HER2 non-amplified primary tumors and HER2 positive or EGFR positive CTCs (ClinicalTrials.gov NCT00820924). The clinical utility of CTCs or HER2-positive CTCs in early BC for patient stratification and as biomarkers to predict benefit from secondary adjuvant treatment interventions with agents like biphosphonates or anti HER2 agents should be prospectively tested.

## Supporting Information

Table S1 *HER2 Intensity CellSearch®* of 30 Circulating Tumor Cells (CTCs) by 3 independent readers.
(DOC)

Table S2 HER2 status on the primary tumor and on Circulating Tumor Cells (CTCs) (only CTC-positive patients are included).
(DOC)
