# Supplementary material for: HER2-Positive Circulating Tumor Cells in Breast Cancer
Source: PLoS One. 2011 Jan 10;6(1):e15624. doi: 10.1371/journal.pone.0015624 (PMC3018524; doi:10.1371/journal.pone.0015624)
Supplement: Table S1 — HER2 Intensity CellSearch® of 30 Circulating Tumor Cells (CTCs) by 3 independent readers. (DOC) [file pone.0015624.s001.doc]

**Table S1.**

| **Patient ID** | **Event (CTC)** | ***HER2 intensity CellSearch®*** | | |
| --- | --- | --- | --- | --- |
|  |  | **Reader 1** | **Reader 2** | **Reader 3** |
| 4 | 12 | 2.1 | 2.6 | 2.5 |
| 14 | 71 | 7.1 | 5.2 | 6.3 |
| 45 | 45 | 1.8 | 2.0 | 2.0 |
| 50 | 2 | 2.3 | 2.4 | 2.8 |
| 50 | 25 | 3.2 | 3.1 | 3.5 |
| 50 | 122 | 2.5 | 2.8 | 2.8 |
| 51 | 88 | 1.7 | 1.6 | 1.8 |
| 85 | 42 | 62.1 | 78.1 | 86.9 |
| 89 | 25 | 3.0 | 3.3 | 4.5 |
| 93 | 26 | 21.3 | 34.9 | 35.2 |
| 125 | 114 | 5.9 | 6.1 | 6.0 |
| 147 | 42 | 2.2 | 2.1 | 3.1 |
| 147 | 112 | 2.6 | 2.7 | 3.8 |
| 209 | 2 | 2.8 | 2.7 | 2.9 |
| 209 | 6 | 2.0 | 2.2 | 2.2 |
| 209 | 8 | 3.3 | 3.0 | 3.1 |
| 209 | 23 | 2.4 | 2.4 | 2.5 |
| 209 | 25 | 2.1 | 2.0 | 2.2 |
| 209 | 104 | 2.7 | 2.9 | 2.7 |
| 209 | 120 | 3.9 | 3.8 | 4.2 |
| 209 | 137 | 3.0 | 3.7 | 4.2 |
| 237 | 33 | 2.4 | 2.4 | 2.6 |
| 237 | 35 | 3.1 | 3.2 | 3.3 |
| 237 | 47 | 2.3 | 2.5 | 2.6 |
| 237 | 58 | 2.9 | 3.1 | 3.4 |
| 237 | 65 | 2.6 | 2.4 | 2.8 |
| 246 | 6 | 3.1 | 3.0 | 3.3 |
| 246 | 8 | 5.5 | 5.2 | 5.9 |
| 246 | 16 | 5.8 | 4.9 | 5.2 |
| 246 | 20 | 1.7 | 1.7 | 1.9 |
