# Supplementary material for: HER2-Positive Circulating Tumor Cells in Breast Cancer
Source: PLoS One. 2011 Jan 10;6(1):e15624. doi: 10.1371/journal.pone.0015624 (PMC3018524; doi:10.1371/journal.pone.0015624)
Supplement: Table S2 — HER2 status on the primary tumor and on Circulating Tumor Cells (CTCs) (only CTC-positive patients are included). (DOC) [file pone.0015624.s002.doc]

**Table S2.**

| **Patient ID** | **Stage** | **HER2 Status**  **Primary Tumor** | **CTCs*** | **HER2-positive CTCs*** |
| --- | --- | --- | --- | --- |
| 85 | LCIS | Negative | 1 | 1 |
| 88 | DCIS | Positive | 1 | 1 |
| 90 | DCIS | Negative | 1 | 0 |
| 190 | DCIS | Negative | 1 | 0 |
| 257 | DCIS | Unknown | 1 | 1 |
| 151 | DCIS | Negative | 2 | 0 |
| 13 | M0 BC | Positive | 1 | 1 |
| 31 | M0 BC | Negative | 1 | 1 |
| 93 | M0 BC | Positive | 1 | 1 |
| 125 | M0 BC | Negative | 1 | 1 |
| 222 | M0 BC | Negative | 1 | 1 |
| 4 | M0 BC | Negative | 3 | 0 |
| 123 | M0 BC | Negative | 1 | 0 |
| 206 | M0 BC | Negative | 1 | 0 |
| 231 | M0 BC | Negative | 1 | 1 |
| 234 | M0 BC | Negative | 1 | 0 |
| 260 | M0 BC | Positive | 1 | 1 |
| 246 | M0 BC | Positive | 4 | 3 |
| 14 | M1 BC | Negative | 1 | 1 |
| 42 | M1 BC | Negative | 1 | 1 |
| 63 | M1 BC | Negative | 1 | 0 |
| 226 | M1 BC | Negative | 1 | 1 |
| 251 | M1 BC | Positive | 1 | 0 |
| 256 | M1 BC | Negative | 1 | 0 |
| 89 | M1 BC | Negative | 2 | 2 |
| 205 | M1 BC | Negative | 5 | 0 |
| 224 | M1 BC | Negative | 6 | 1 |
| 229 | M1 BC | Positive | 7 | 1 |
| 53 | M1 BC | Negative | 9 | 0 |
| 250 | M1 BC | Negative | 9 | 3 |
| 44 | M1 BC | Negative | 11 | 0 |
| 212 | M1 BC | Negative | 13 | 1 |
| 39 | M1 BC | Negative | 14 | 0 |
| 45 | M1 BC | Negative | 14 | 0 |
| 51 | M1 BC | Negative | 16 | 1 |
| 237 | M1 BC | Negative | 28 | 5 |
| 252 | M1 BC | Negative | 31 | 0 |
| 50 | M1 BC | Negative | 75 | 3 |
| 147 | M1 BC | Negative | 75 | 2 |
| 209 | M1 BC | Negative | 193 | 42 |
| 43 | M1 BC | Negative | 640 | 2 |

DCIS: Ductal Carcinoma in situ, LCIS: Lobular Carcinoma in situ, M0 BC: Non-metastatic Breast Cancer, M1 BC: Metastatic Breast Cancer.

*Number of CTCs or HER2-positive CTCs per 22.5mL of blood (DCIS, LCIS, M0 BC) or per 7.5mL of blood (M1 BC).
